# DBC1 regulates Wnt/β-catenin-mediated expression of MACC1, a key regulator of cancer progression, in colon cancer

**DOI:** 10.1038/s41419-018-0899-9

**Published:** 2018-08-06

**Authors:** Hwa Jin Kim, Sue Jin Moon, Seok-Hyung Kim, Kyu Heo, Jeong Hoon Kim

**Affiliations:** 1 Department of Health Sciences and Technology, Samsung Advanced Institute for Health Sciences and Technology, Sungkyunkwan University, Seoul, 06351 Korea; 2 Department of Biomedical Sciences, Samsung Biomedical Research Institute, Samsung Medical Center, Seoul, 06351 Korea; 3 Department of Pathology, Samsung Medical Center, Sungkyunkwan University School of Medicine, Seoul, 06351 Korea; 4 Department of Clinical Research, Dongnam Institute of Radiological and Medical Sciences, Busan, 46033 Korea

## Abstract

Metastasis-associated in colon cancer 1 (MACC1) has been reported to be overexpressed in multiple cancers and promote proliferation, metastasis, cancer stem cell-like properties, and drug resistance of cancer cells. Despite its significance and the considerable knowledge accumulated on the function of MACC1 in various types of human malignancies, regulatory mechanisms underlying MACC1 expression remain unclear. Here we report that MACC1 is a direct target of Wnt/β-catenin signaling pathway in colon cancer cells and that DBC1 functions as a coactivator for Wnt-mediated MACC1 expression by promoting the activity of a LEF1/β-catenin-dependent enhancer located in intron 1 of MACC1 gene. DBC1 is required for LEF1/β-catenin complex formation on the MACC1 enhancer and for long-distance enhancer-promoter interaction of the MACC1 locus. MACC1 expression was increased in colonosphere cells compared to adherent colon cancer cells, and DBC1 overexpression further increased MACC1 expression in colonospheres and promoted sphere-forming abilities of colon cancer cells and drug resistance of colonospheres. Importantly, expressions of MACC1 and DBC1 are positively correlated with each other, upregulated in high-risk groups of colorectal cancer patients, and associated with poor survival. Our results establish MACC1 as a transcriptional target of Wnt/β-catenin signaling and suggest that DBC1 plays a key role in colorectal cancer progression through Wnt/β-catenin-MACC1 signaling axis.

## Introduction

Wnt/β-catenin signaling plays a crucial role in a wide range of developmental and oncogenic processes^1–5^. Upon Wnt stimulation, β-catenin is stabilized by escaping from GSK3β-mediated phosphorylation-dependent degradation, and accumulated β-catenin translocates into the nucleus and activates Wnt target gene transcription through interactions with TCF/LEF family transcription factors on Wnt responsive elements (WREs) in target enhancers^1–5^. Aberrant activation of the Wnt/β-catenin signaling cascade is involved in the initiation and progression of numerous human cancers, including colorectal cancer (CRC), and contributes to maintenance of cancer stem cells (CSCs) and chemoresistance in colon cancer cells^1–5^. However, little is known about detailed mechanism underlying the regulation of β-catenin activity and which β-catenin target genes are essential in colon cancer progression and metastasis.

Deleted in breast cancer (DBC1/CCAR2) is a multifunctional protein involved in a variety of physiological and pathological processes including apoptosis and tumorigenesis^6^. We and others have recently shown that DBC1 plays a key role in multiple oncogenic signaling pathways by acting as a transcriptional coactivator for estrogen receptor, PEA3/ETV4, LEF1-β-catenin, androgen receptor, and androgen receptor variant 7 and by functioning as an inhibitor of epigenetic modifiers such as SIRT1, HDAC3, SUV39H1, MDM2, and CHIP^7–14^. In colon cancer cells, DBC1 functions as a coactivator of LEF1-β-catenin-mediated transcription by protecting β-catenin from SIRT1-mediated deacetylation and repression^9^. In addition, DBC1 not only promotes the expression of a Wnt/β-catenin-inducible transcription factor PROX1, which plays a critical role in CRC progression, but also serves as a coactivator of PROX1, suggesting DBC1 as a key regulator in CRC progression driven by Wnt/β-catenin-PROX1 signaling^9^.

Metastasis-associated in colon cancer 1 (MACC1), originally identified as a critical regulator of the HGF-MET signaling, is frequently overexpressed in CRC and promotes proliferation, epithelial-mesenchymal transition (EMT), metastasis, CSC-like properties, and chemoresistance of colon cancer cells by acting as a transcriptional activator of c-MET and other cancer-related genes including SPON2, OCT4, NANOG, and MDR1/ABCB1^15–19^. Accumulating studies suggest that MACC1 is a prognostic and metastatic biomarker for colon cancer and various other cancers^15,17,19–22^. A recent study showed a significant positive correlation between β-catenin and MACC1 expression in CRC and that MACC1 positively regulates β-catenin signaling by up-regulating β-catenin expression^23^. However, it remains unknown how the expression of MACC1 is regulated in colon cancer cells. Here we report MACC1 as a direct target of Wnt/β-catenin signaling, and a novel role of DBC1 in CRC progression through activating Wnt/β-catenin-MACC1 signaling axis.

## Results

### MACC1 expression is upregulated by Wnt/β-catenin signaling

**Fig. 1:**
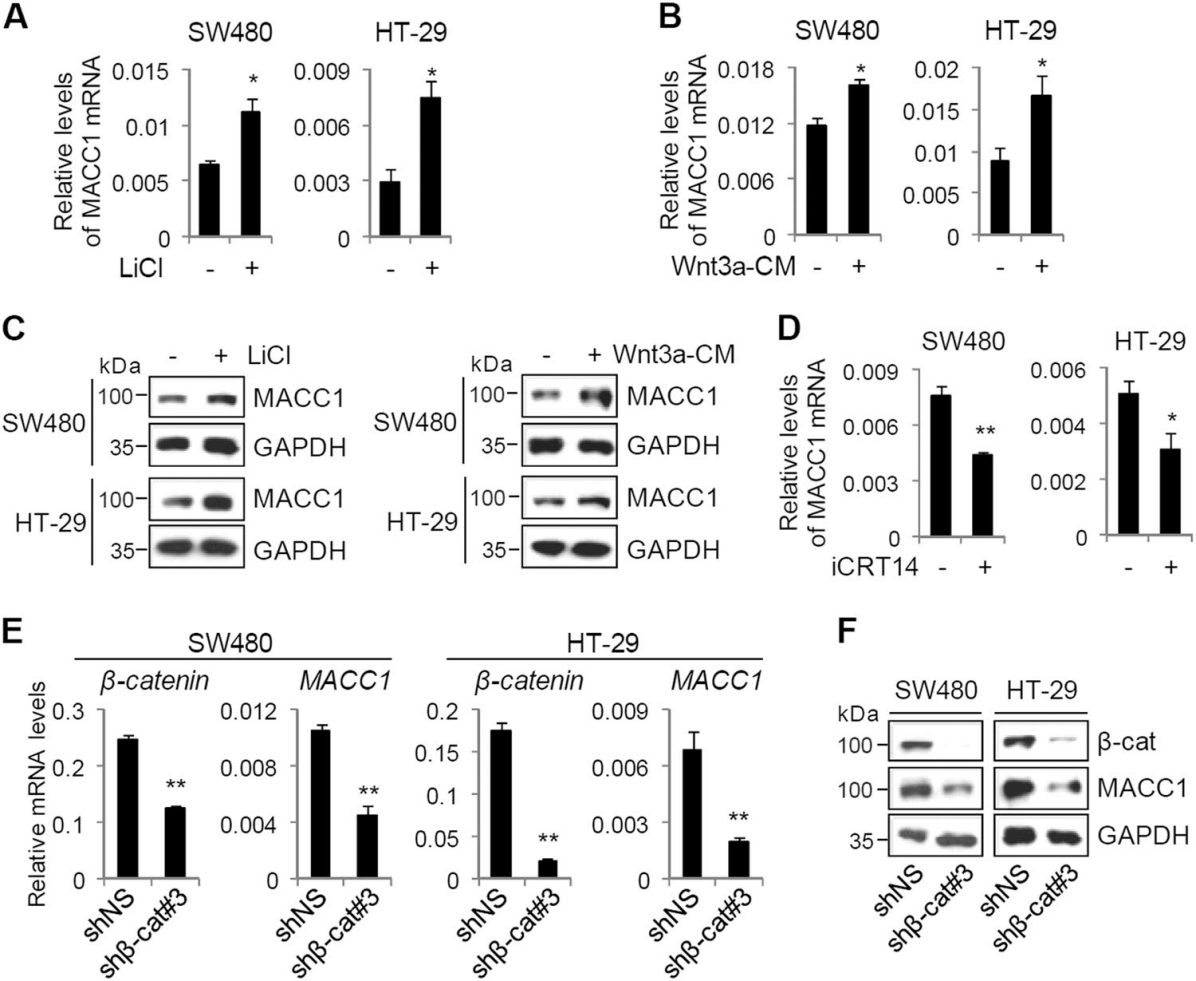
MACC1 is a target of Wnt/β-catenin signaling. **a**–**c** SW480 and HT-29 cells were treated with 20 mM LiCl or Wnt3a-CM for 48 h. MACC1 mRNA and protein levels were examined by qRT-PCR (**a**, **b**) and immunoblot (**c**). Data are means ± s.d. (*n* = 3). **d** SW480 and HT-29 cells were treated with 50 µM iCRT14 for 24 h. MACC1 mRNA levels were examined by qRT-PCR. Data are means ± s.d. (*n* = 3). **e**, **f** SW480 and HT-29 cells were infected with lentiviruses-expressing shNS or shβ-catenin#3. The mRNA and protein levels of β-catenin and MACC1 were examined by qRT-PCR (**e**) and immunoblot (**f**). Data are means ± s.d. (*n* = 3). *P < 0.05 and **P < 0.01

To investigate whether MACC1 expression is regulated by Wnt/β-catenin signaling pathway, we treated colon cancer cells (SW480 and HT-29) with either LiCl, a GSK3β inhibitor, or Wnt3a-conditioned media (CM), and monitored the expression levels of MACC1 by quantitative real-time reverse transcription-PCR (qRT-PCR) and immunoblot analysis. Both LiCl and Wnt3a-CM increased the expression levels of MACC1 mRNA and protein (Fig. 1a–c), suggesting that MACC1 expression is regulated by Wnt signaling. We next assessed the effects of iCRT14, a β-catenin-TCF complex inhibitor, and β-catenin knockdown on MACC1 expression in colon cancer cells. iCRT14 decreased MACC1 expression in colon cancer cells (Fig. 1d). Consistent with these results, depletion of β-catenin reduced both mRNA and protein levels of MACC1 in colon cancer cells (Fig. 1e, f). Similar results were observed with two additional shRNAs targeting different regions of β-catenin mRNA in SW480 cells (Supplementary Fig. S1a, b). These results suggest that the expression of MACC1 is transcriptionally regulated by Wnt/β-catenin signaling in colon cancer cells.

### DBC1 is required for β-catenin-mediated MACC1 expression

**Fig. 2:**
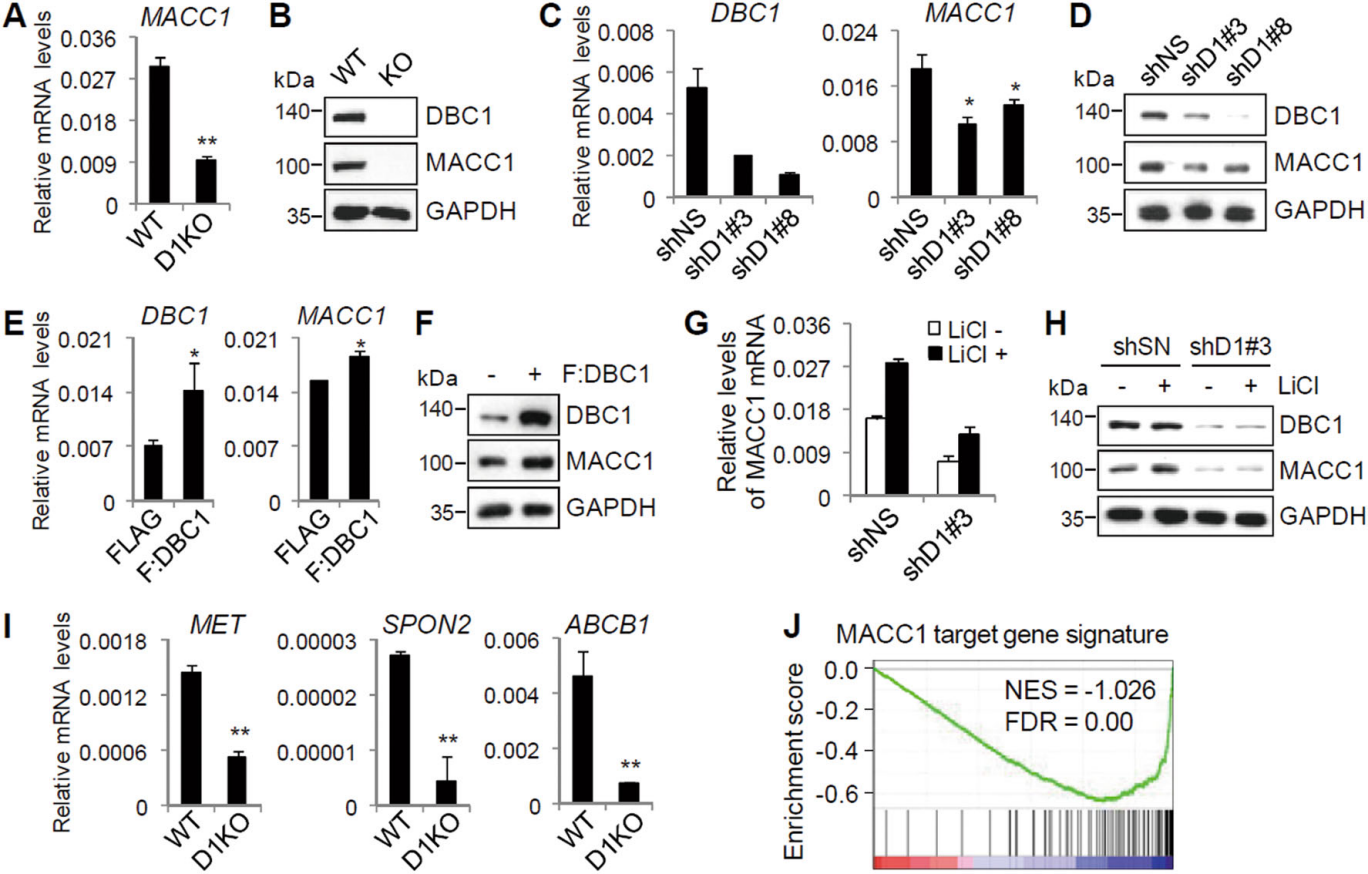
DBC1 positively regulates Wnt/β-catenin-mediated MACC1 expression. **a**, **b** MACC1 mRNA and protein levels in WT and DBC1 KO SW480 cells were examined by qRT-PCR (**a**) and immunoblot (**b**). Data are means ± s.d. (*n* = 3). **c**–**f** HT-29 cells were infected with lentiviruses-expressing shNS or shDBC1s (**c**, **d**) or lentiviruses-expressing FLAG or FLAG-DBC1 (**e**, **f**). DBC1 and MACC1 mRNA and protein levels were examined by qRT-PCR and immunoblot. Data are means ± s.d. (*n* = 3). **g**, **h** HT-29 cells were infected with lentiviruses-expressing shNS or shDBC1#3, serum-starved, and treated with 20 mM LiCl. MACC1 mRNA levels were examined by qRT-PCR (**g**), and protein levels were monitored by immunoblot using the indicated antibodies (**h**). Data are means ± s.d. (*n* = 3). **i** Validation of DBC1-regulated MACC1 target genes. Total RNAs from SW480 WT and DBC1 KO cells were examined by qRT-PCR analysis with primers specific for the indicated mRNAs. Data are means ± s.d. (*n* = 3). **j** GSEA profile of the MACC1 target gene signature among DBC1-regulated genes in SW480 cells. *P < 0.05 and **P < 0.01

Given our recent report that DBC1 is a critical regulator of Wnt/β-catenin signaling in CRC^9^, we next tested the possibility that DBC1 may also contribute to β-catenin-mediated upregulation of MACC1 in colon cancer cells. Interestingly, from our previous microarray data, we found that MACC1 expression was downregulated in DBC1 knockout (KO) SW480 cells compared with wild-type cells^9^. We thus validated microarray results with qRT-PCR and immunoblot analysis. Both mRNA and protein levels of MACC1 were reduced by DBC1 KO in SW480 cells (Fig. 2a, b). Similar results were also observed in DBC1-depleted HT-29 cells (Fig. 2c, d). In contrast, DBC1 overexpression increased mRNA and protein levels of MACC1 in HT-29 cells (Fig. 2e, f). In addition, DBC1 depletion greatly reduced LiCl-induced expression of MACC1 (Fig. 2g, h), indicating that DBC1 is required for Wnt/β-catenin-mediated expression of MACC1 in colon cancer cells. Interestingly, comparison of DBC1 KO SW480 microarray data^9^ with MACC1 overexpression SW480 microarray result^17^ revealed 74 shared target genes between DBC1 and MACC1 (Supplementary Fig. S2). We selected seven genes including c-Met, SPON2, and ABCB1/MDR1, which have been shown to play important roles in metastasis and drug resistance in numerous types of cancer^15,17,18,24–27^, and validated their expression levels by qRT-PCR (Fig. 2i and Supplementary Fig. S3). Gene set enrichment analysis (GSEA) using the MACC1 target gene signature revealed a significant reduction in the expression of MACC1 target genes in DBC1 KO cells (Fig. 2j), suggesting that their expression is reduced as a result of DBC1 KO-mediated downregulation of MACC1.

### DBC1 cooperates synergistically with β-catenin to activate MACC1 enhancer activity

**Fig. 3:**
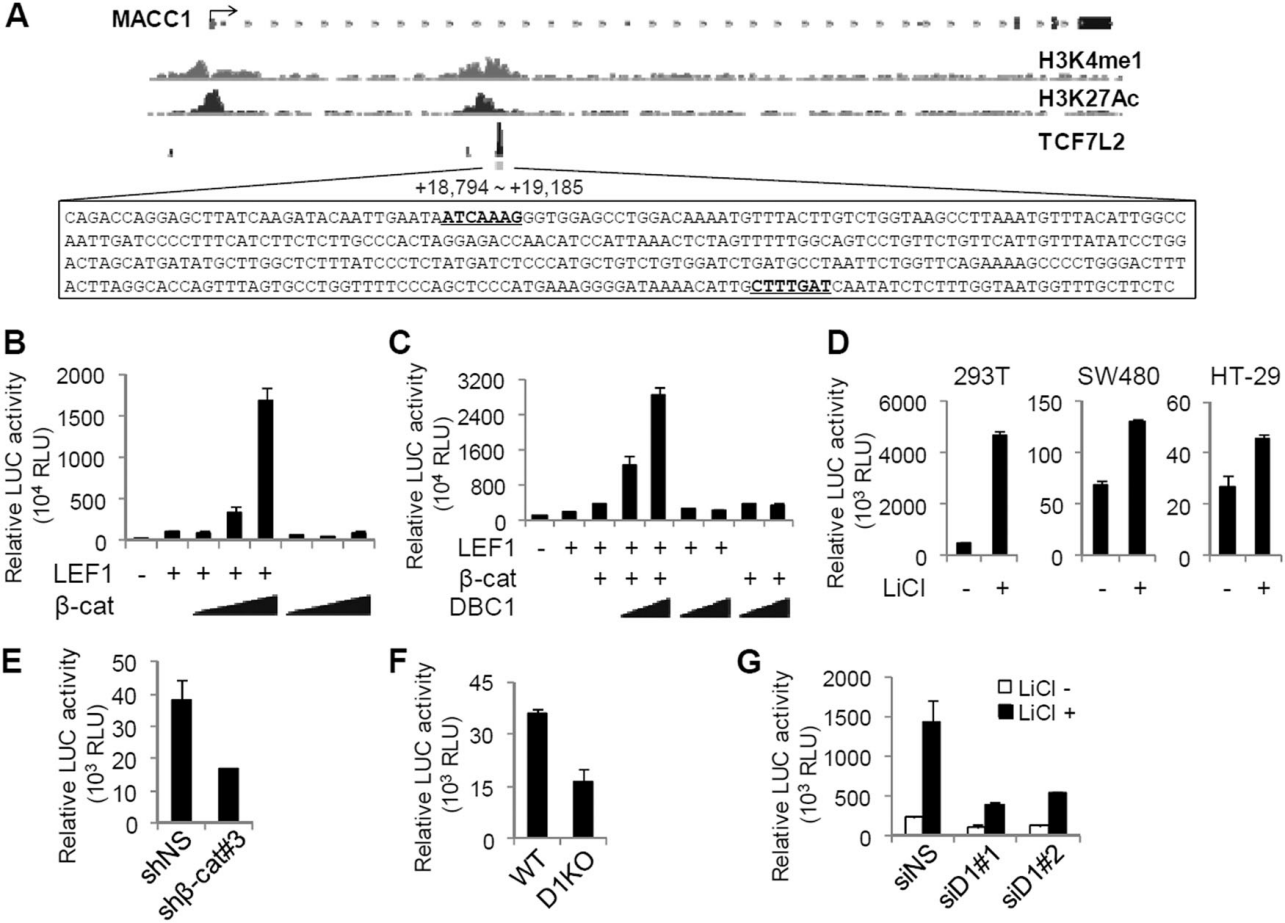
DBC1 and β-catenin synergistically activate the MACC1 enhancer. **a** ChIP-seq tracks for H3K4me1, H3K27Ac, and TCF4/TCF7L2 at the MACC1 locus. The TCF4-binding region is indicated by a gray box, and the DNA sequence of the region is shown. Predicted TCF4-binding sites are underlined. **b**, **c** The 293T cells were transfected with MACC1.enh-TA-LUC reporter along with pSG5.HA-LEF1 and increasing amounts of pSG5.HA-β-catenin or pSG5.HA-DBC1, as indicated, and harvested for luciferase assays. Data are means ± s.d. (*n* = 3). **d** The 293T, SW480, and HT-29 cells were transfected with MACC1.enh-TA-LUC reporter and treated with 20 mM LiCl. Data are means ± s.d. (*n* = 3). **e**, **f** MACC1.enh-TA-LUC reporter activity in SW480 cells infected with lentiviruses-expressing shNS or shβ-catenin (**e**) or in SW480 WT and DBC1 KO cells (**f**). Data are means ± s.d. (*n* = 3). **g** 293T cells transfected with MACC1.enh-TA-LUC and indicated siRNAs against DBC1 were treated with 20 mM LiCl. Data are means ± s.d. (*n* = 3)

To identify functional WREs that control MACC1 expression, we analyzed publicly available ChIP-seq datasets from ENCODE (UCSC genome browser) and found a TCF4/TCF7L2-binding region located in MACC1 gene intron 1, approximately 19 kb downstream of the transcription start site (TSS) (Fig. 3a). Further sequence analysis revealed the presence of two putative TCF4/LEF1 binding sites (WRE, 5′-CTTTGWW-3′) within this region (Fig. 3a). Interestingly, active enhancer marks, histone H3 lysine 4 mono-methylation (H3K4me1) and histone H3 lysine 27 acetylation (H3K27Ac), are also enriched in this region (Fig. 3a), suggesting that this region might function as an enhancer for the MACC1 gene transcription. Indeed, in luciferase assays using a reporter containing two putative WREs, LEF1 activated the reporter activity, and β-catenin further enhanced the reporter activity in a dose-dependent and LEF1-dependent manner (Fig. 3b). Furthermore, co-expression of β-catenin and DBC1 synergistically enhanced LEF1 activity (Fig. 3c). The coactivator activity of DBC1 completely depended on the presence of β-catenin (Fig. 3c), suggesting that DBC1 enhances β-catenin-dependent LEF1 transcriptional activity as a secondary coactivator.

Consistent with the qRT-PCR data (Figs. 1, 2), LiCl increased the MACC1 enhancer-reporter activity in 293T, SW480, and HT-29 cells (Fig. 3d). In contrast, depletion of β-catenin or DBC1 KO decreased the reporter activity in colon cancer cells (Fig. 3e, f and Supplementary Fig. S1c). Moreover, DBC1 depletion greatly inhibited the reporter activity induced by LiCl (Fig. 3g). Taken together, these results suggest that the TCF4/LEF1 binding region located in intron 1 contains transcriptional enhancer elements responsive to Wnt/β-catenin signaling and that DBC1 cooperates synergistically with β-catenin to enhance LEF1-mediated transcriptional activation of the MACC1 enhancer.

### DBC1 is required for efficient binding of LEF1/TCF4 to the MACC1 enhancer

**Fig. 4:**
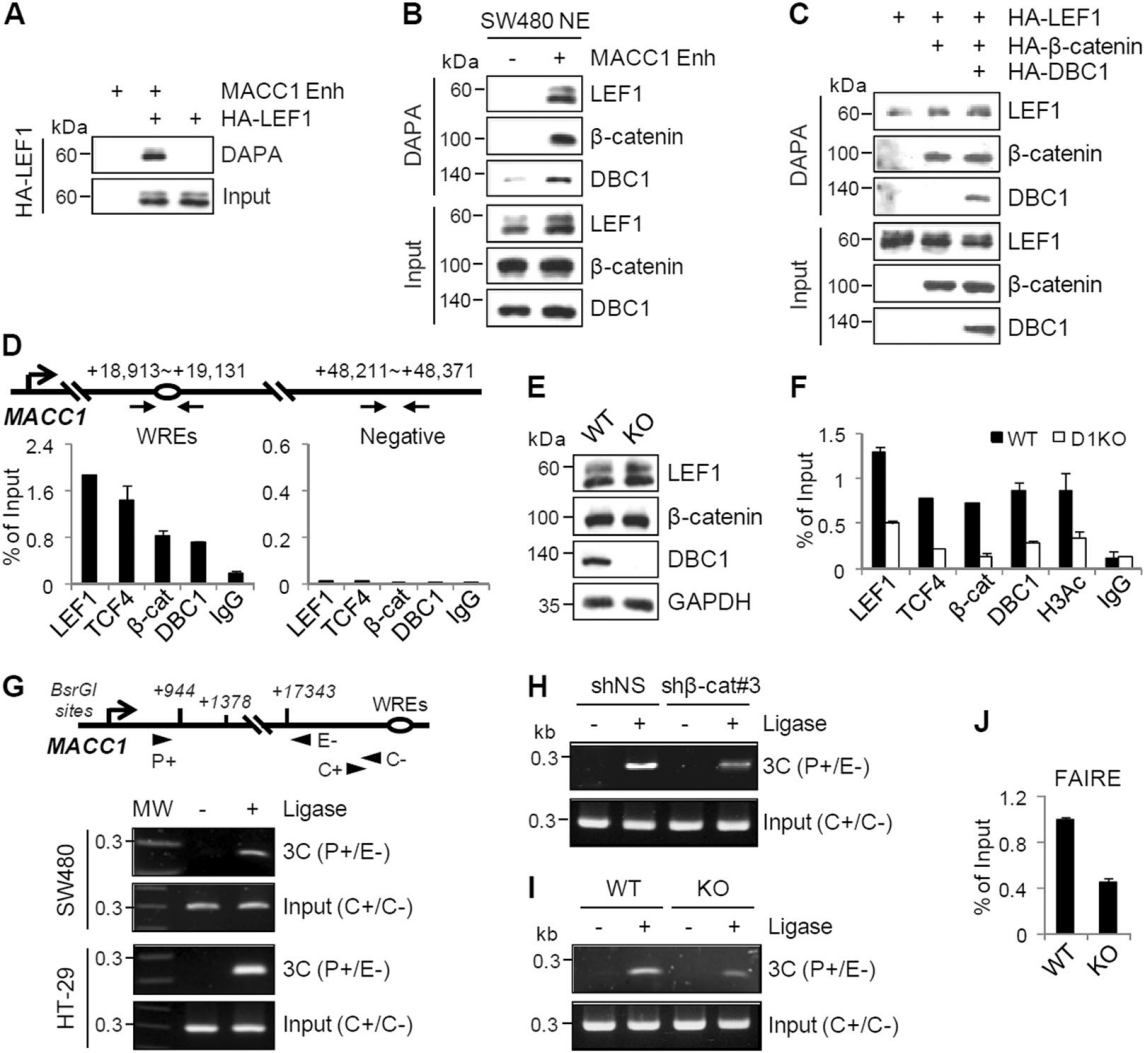
Requirement of DBC1 for efficient binding of LEF1 to the MACC1 enhancer and for chromatin looping between the enhancer and promoter of the MACC1 gene. **a** DAPA was performed using a biotinylated MACC1 enhancer probe and recombinant HA-LEF1 protein. DNA-bound LEF1 were precipitated by Streptavidin beads and analyzed by immunoblot. **b**, **c** DAPA using SW480 cell nuclear extract (NE) (**b**) or 293T cell lysates transfected with the indicated expression vectors (**c**) was performed as described above. **d** ChIP assays. After immunoprecipitation of sheared chromatin from SW480 cells with the indicated antibodies, qPCR analyses were performed using primers specific for the indicated regions of the MACC1 gene. The results are shown as the percentage of input and are means ± s.d. (*n* = 3). **e**, **f** Protein levels in SW480 WT and DBC1 KO cells were monitored by immunoblot (**e**), and ChIP assays with the indicated antibodies were performed as described above (**f**). **g** The 3C experiments. Cross-linked chromatins from SW480 and HT-29 cells were digested with BsrGI, diluted, and ligated. After reverse-crosslinking, equal amounts of 3C DNA were amplified by PCR using primer pairs P+ and E- (3C) or C+ and C- (Input). **h**, **i** 3C assays were performed using chromatin from HT-29 cells expressing shNS or shβ-catenin (**h**) or chromatin from SW480 WT and DBC1 KO cells (**i**) as described above. **j** FAIRE assays were performed in SW480 WT and DBC1 KO cells, and FAIRE DNA samples were analyzed by qPCR

To determine whether LEF1 binds directly to the identified MACC1 enhancer, we performed DNA affinity precipitation assays (DAPA). The 293T cell extracts transfected with LEF1 were incubated with a biotinylated MACC1 enhancer DNA probes, and the DNA-protein complexes were pulled down with Streptavidin beads and analyzed by immunoblot. As shown in Fig. 4a, LEF1 was precipitated with the enhancer probe, indicating that LEF1 binds directly to the MACC1 enhancer. DAPA using nuclear extracts from SW480 also showed that endogenous LEF1 bound to the MACC1 enhancer and that β-catenin and DBC1 were also associated with the enhancer (Fig. 4b). In addition, LEF1 binding to the enhancer was increased by enforced expression of β-catenin and further enhanced by co-overexpression of DBC1 (Fig. 4c), suggesting that association of β-catenin and DBC1 with LEF1 stabilizes the interaction between the MACC1 enhancer and LEF1.

To investigate the in vivo association of the endogenous LEF1/TCF4, β-catenin, and DBC1 with the native MACC1 enhancer, we next conducted chromatin immunoprecipitation (ChIP) assays in SW480 cells. LEF1, TCF4, β-catenin, and DBC1 were recruited to the MACC1 enhancer, but not to a control region lacking a LEF1/TCF4-binding site (Fig. 4d), suggesting that they are directly involved in the transcriptional control of the MACC1 gene. To assess the role of DBC1 in transcription complex assembly on the MACC1 enhancer, we repeated ChIP assays in DBC1 KO SW480 cells. DBC1 loss had no effect on protein levels of LEF1 and β-catenin, but greatly reduced their occupancy at the MACC1 enhancer (Fig. 4e, f). Together, these results suggest that DBC1 is required for efficient binding of LEF1/TCF4 to the MACC1 enhancer, and thereby facilitates subsequent recruitment of β-catenin to the enhancer.

### β-catenin and DBC1 are required for long-range chromatin interaction between the enhancer and promoter of the MACC1 gene

The mechanism of enhancer action is thought to involve chromatin looping, which may bring the distal enhancer in close proximity to the target promoter^28^. We therefore hypothesized that the distal MACC1 enhancer located 19 kb downstream of the TSS physically associates with the MACC1 promoter through chromatin looping and tested such physical communications using chromosome conformation capture (3C) technique in SW480 and HT-29 cells. The specific 3C-PCR product was amplified in a ligase-dependent manner (Fig. 4g), indicating a specific physical interaction between the MACC1 enhancer and promoter. DNA sequencing of 3C-PCR products confirmed their association (Supplementary Fig. S4). In addition, β-catenin depletion and DBC1 KO reduced the MACC1 enhancer-promoter loop formation (Fig. 4h, i and Supplementary Fig. S5), indicating that both β-catenin and DBC1 participate in bridging between the distal enhancer and promoter of the MACC1 gene.

Furthermore, formaldehyde-assisted isolation of regulatory elements (FAIRE) analysis revealed that DBC1 KO caused a significant reduction in chromatin accessibility at the MACC1 enhancer region (Fig. 4j). This is consistent with our ChIP data (Fig. 4f), showing that histone H3 acetylation levels at the MACC1 enhancer are reduced by DBC1 KO. Collectively, these results suggest a critical role for DBC1 in regulating chromatin architecture of the MACC1 gene by mediating long-range chromatin interactions and maintaining open chromatin.

### DBC1 promotes MACC1 expression in colonospheres and CSC-like properties of colon cancer cells

**Fig. 5:**
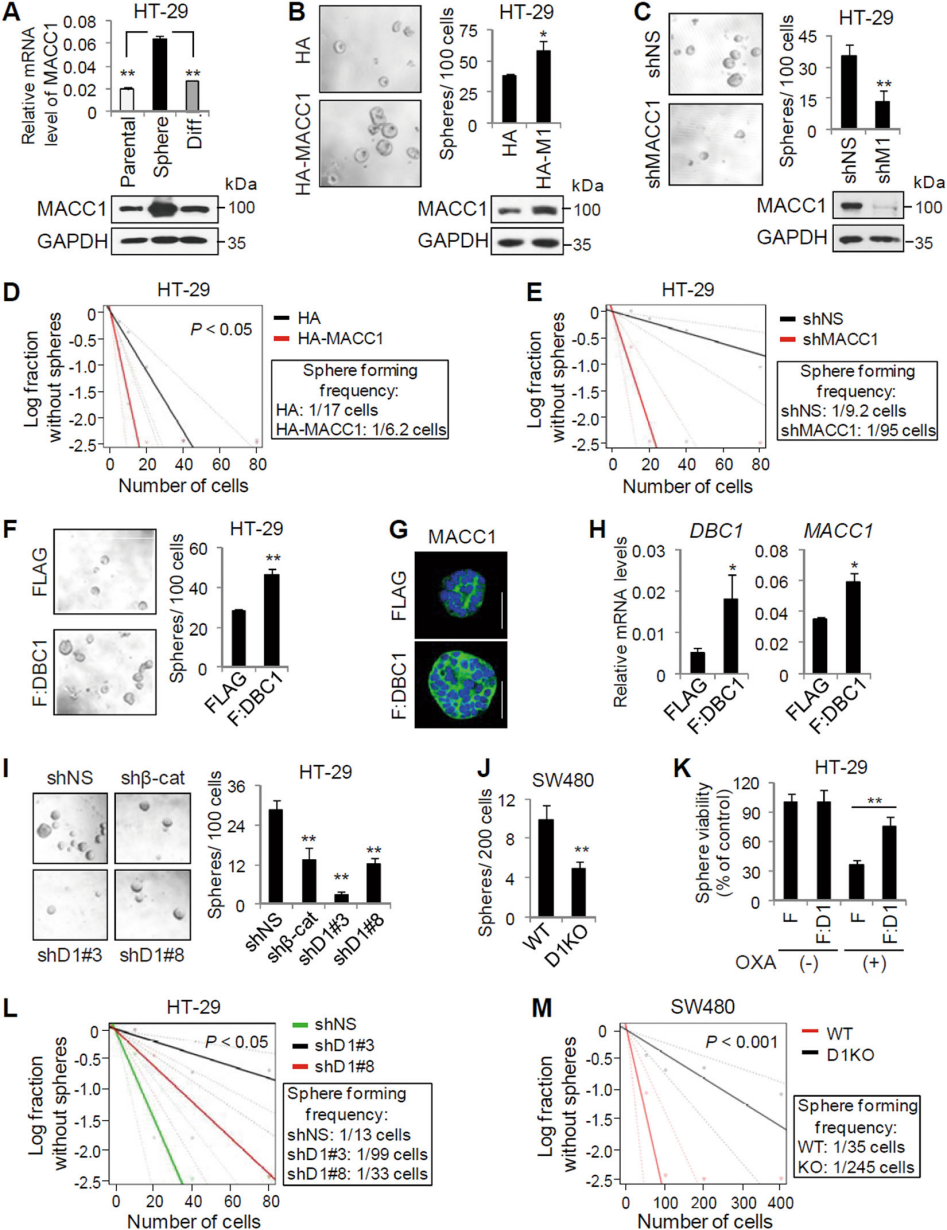
DBC1 is required for CSC-like properties of colon cancer cells and for MACC1 expression in colon CSC-like cells. **a** Comparison of MACC1 mRNA and protein levels in parental HT-29 cells, HT-29 colonospheres (sphere), and their differentiated (diff.) cells. MACC1 mRNA and protein levels were determined by qRT-PCR (data are means ± s.d. (*n* = 3)) and immunoblot, respectively. **b**, **c** Colonosphere assays using HT-29 cells expressing HA or HA-MACC1 (**b**) and using HT-29 cells infected with lentiviruses-expressing shNS or shMACC1 (**c**). Data are means ± s.d. (*n* = 6). Protein levels were monitored by immunoblot. **d**, **e** LDA using HT-29 cells expressing HA or HA-MACC1 **(d)** and using HT-29 cells infected with lentiviruses-expressing shNS or shMACC1 **(e)**. **f**–**h** Colonosphere assays using HT-29 cells infected with lentiviruses-expressing FLAG or FLAG-DBC1 (**f**). Data are means ± s.d. (*n* = 6). Protein and mRNA levels of MACC1 in HT-29 spheres were determined by immunofluorescence (**g**) and qRT-PCR (**h**). **i** Colonosphere assays using HT-29 cells infected with lentiviruses-expressing shNS, shβ-catenin, or shDBC1s as indicated. Data are means ± s.d. (*n* = 6). **j** Colonosphere assays using SW480 WT and DBC1 KO cells. Data are means ± s.d. (*n* = 6). **k** HT-29 colonospheres expressing FLAG or FLAG-DBC1 were treated with vehicle or 10 µM oxaliplatin. Data are presented as percent of control (vehicle-treated cells). Data are means ± s.d. (*n* = 6). **l**, **m** LDA using HT-29 cells expressing shNS or shDBC1s (**l**) and using SW480 WT or DBC1 KO cells (**m**). *P < 0.05 and **P < 0.01

Given that MACC1 has been shown to induce CSC-like characteristics including self-renewal capability and drug resistance^16,18,22^, we next investigated the role of DBC1 in the regulation of MACC1 expression in colonospheres and in CSC-like properties of colon cancer cells. Compared with monolayer culture, HT-29 cells grown in sphere culture, enriching for CSC-like cells^29,30^, had higher expression of MACC1, and MACC1 expression was greatly reduced in sphere-derived differentiated HT-29 cells to the level in parental HT-29 cells (Fig. 5a). These results indicate that MACC1 is preferentially upregulated in CSC-like colonospheres. MACC1 overexpression increased, while its knockdown decreased, both the size and number of colonospheres (Fig. 5b, c). To elucidate the role of MACC1 in colonospheres formation, we next performed limiting dilution assays (LDA). MACC1 overexpression significantly increased the frequency of colonosphere-forming cells (Fig. 5d), whereas its depletion reduced the colonosphere-forming ability of HT-29 cells (Fig. 5e), indicating a critical role of MACC1 in self-renewal capacity of colon CSC-like cells.

In addition, overexpression of DBC1 also increased the number and size of colonospheres as well as both protein and mRNA levels of MACC1 in colonospheres (Fig. 5f–h). In contrast, colonosphere numbers of HT-29 and SW480 cells were reduced by DBC1 depletion or KO (Fig. 5i, j). Furthermore, overexpression of DBC1 decreased the chemosensitivity of HT-29 colonospheres to oxaliplatin (Fig. 5k). These results suggest that DBC1 contributes to CSC-like properties of colon cancer cells, probably through promoting MACC1 expression. Consistent with a functional role of DBC1 in colonosphere formation, DBC1 depletion caused 2.5 ~ 7.6-fold decrease in sphere-initiating cell frequency in HT-29 cells (Fig. 5l), and similar result was also observed in DBC1 KO SW480 cells (a sevenfold decrease in sphere-initiating cell frequency) (Fig. 5m). Taken together, these results indicate that DBC1 is required for the self-renewal of colon CSC-like cells.

### High expression of both DBC1 and MACC1 are associated with poor prognosis of colon cancer

**Fig. 6:**
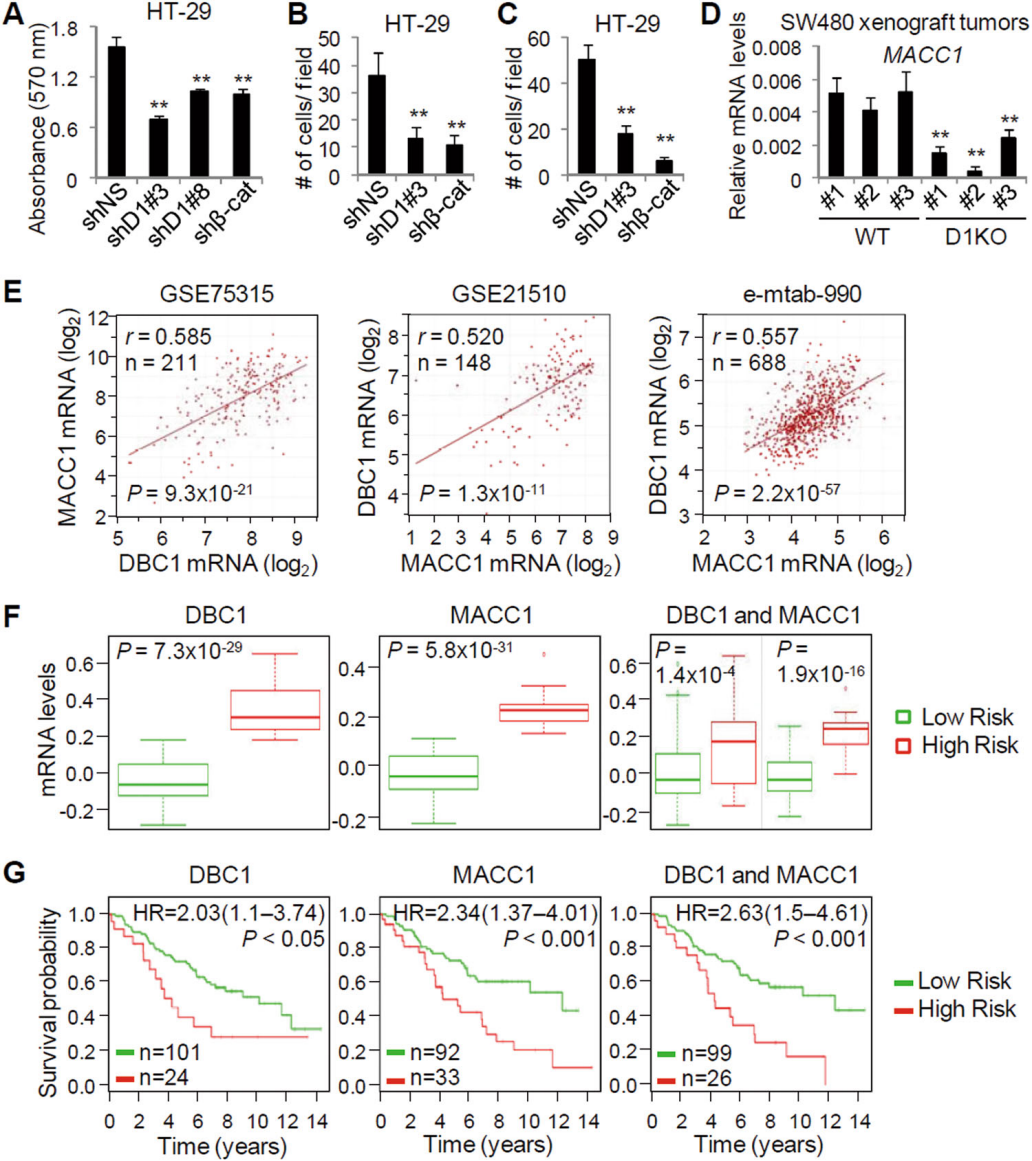
DBC1 and MACC1 expression in colon cancer patients predicts clinical outcomes. **a**–**c** Cell proliferation (**a**), migration (**b**), and invasion (**c**) assays using HT-29 cells infected with lentiviruses-expressing shNS, shβ-catenin, or shDBC1s as indicated. **d** qRT-PCR analysis of MACC1 expression in SW480 WT and DBC1 KO xenograft tumors. Data are means ± s.d. (*n* = 3). **e** Correlation analysis of MACC1 and DBC1 expression in colon cancer. Plotted data are log_2_ mRNA expression from GSE75315, GSE21510, and e-mtab-990 datasets. **f**, **g** DBC1 and MACC1 correlate with poor prognosis of colon cancer patients. Box-plots and Kaplan-Meier survival curves were generated with SurvExpress biomarker validation tool using a colon cancer dataset (GSE28722). Expression levels of DBC1, MACC1, and both genes stratified by risk groups (**f**) and Kaplan-Meier survival curves of Cox analysis for the colon cancer dataset stratified by maximized DBC1, MACC1, and both gene expression risk groups (**g**). **P < 0.01

Our recent study has shown that DBC1 is required for in vitro and in vivo growth of colon cancer cells^9^. Indeed, cell proliferation of HT-29 was inhibited by DBC1 depletion, as well as by β-catenin depletion (Fig. 6a). Similarly, depletion of DBC1 or β-catenin led to reduced migration and invasion of HT-29 cells (Fig. 6b, c), confirming that both DBC1 and β-catenin are required for the growth and metastatic behavior of colon cancer cells. To assess the role of DBC1 in in vivo expression of MACC1, we next performed qRT-PCR and immunohistochemistry analyses using SW480 xenograft tumors obtained from our previous study^9^. Loss of DBC1 reduced mRNA levels of MACC1 in SW480 xenograft tumors (Fig. 6d and Supplementary Fig. S6), suggesting a critical role of DBC1 in in vivo expression of MACC1.

To further validate our finding of DBC1 as a positive regulator of MACC1 expression in CRC, we performed the expression correlation analysis on human CRC gene expression datasets using R2 Genomics Analysis and Visualization Platform (http://r2.amc.nl). MACC1 mRNA expression was significantly correlated with levels of DBC1 in three CRC datasets tested (Fig. 6e), confirming the positive role of DBC1 in MACC1 expression in CRC. We next investigated the clinical relevance of DBC1 and MACC1 expression in CRC using the SurvExpress, a cancer gene expression database with clinical outcomes and an online tool to provide risk assessment of cancer datasets^31^. Cox analysis using the GSE28722 and GSE24551 datasets showed that mRNA levels of MACC1 and DBC1 are significantly upregulated in high-risk groups of CRC patients, and that high expression of MACC1 and DBC1 is associated with poor overall and disease-free survival (Fig. 6f, g and Supplementary Fig. S7). In the MACC1-DBC1 pair, CRC patients with high expression of both genes had a worse outcome than those with low expression of both genes, indicating the tumor promoting role of the DBC1-MACC1 axis in CRC progression.

## Discussion

Metastasis is the most common cause of treatment failure and most lethal characteristic of CRC^20^. Accumulating evidence suggests that MACC1 is a promising biomarker for metastasis and prognosis of CRC and also a therapeutic target in the treatment of CRC^15–18,20–23^. MACC1 is highly expressed in CRC with high metastatic propensity, and the level of circulating MACC1 transcripts in plasma of CRC patients is correlated with unfavorable survival and metastasis^15,32^. In addition to stimulating colon cancer cell proliferation and metastasis, MACC1 promotes chemoresistance and CSC-like properties in colon cancer cells^16,18^. Despite its significance and the considerable knowledge accumulated on the function of MACC1 in various types of human malignancies such as breast, ovarian, lung, hepatocellular, gastric cancers as well as colon cancer^15,33–37^, our understanding on the regulatory mechanism of MACC1 expression has remained very limited. Cancer-specific activation of MACC1 expression is possibly mediated by aberrantly activated cell signaling pathways in cancer cells. Here, we showed MACC1 as a direct target of the canonical Wnt signaling in colon cancer cells. Activation of Wnt signaling stimulated MACC1 expression in colon cancer cells. Conversely, inhibition of the Wnt signaling by either β-catenin depletion or β-catenin inhibitor treatment resulted in reduction in MACC1 expression. We identified an enhancer region in the intron 1 of the MACC1 gene essential for induction by Wnt/β-catenin signaling and further showed that β-catenin enhances MACC1 expression by mediating chromatin looping between the MACC1 enhancer and promoter. Previous studies demonstrated that AP-1, SP1, C/EBP, and YB-1 interacts with the promoter of MACC1 gene and stimulate its transcription^38,39^. In addition, AP-1 transcription factors, including c-Jun and c-Fos, are able to associate with β-catenin and activate β-catenin target gene expression^40^. Thus, it is conceivable that β-catenin bridges LEF1/TCF4 transcription factors bound to distant enhancer to AP-1 bound to the MACC1 promoter.

In this study, we also showed that DBC1 functions as a coactivator for β-catenin-mediated MACC1 expression and cooperates synergistically with β-catenin to activate MACC1 enhancer activity. In line with our previous findings that DBC1 enhances LEF1-β-catenin complex formation on WREs of β-catenin targets including a Wnt-inducible transcription factor PROX1 involved in colon cancer progression^9^, DBC1 increases LEF1-β-catenin interaction on the MACC1 enhancer, and loss of DBC1 reduced the occupancy of LEF1, TCF4, and β-catenin on the MACC1 enhancer. These results, along with the fact that DBC1 is required for the MACC1 enhancer-promoter looping, strongly suggest that DBC1 functions as a critical regulator of Wnt/β-catenin-mediated transcription of MACC1 by regulating chromatin architecture of the MACC1 locus and LEF1/TCF4-β-catenin complex assemble on the MACC1 enhancer.

Negative and positive feedback regulatory loops are a critical mechanism to precisely control cell signaling. Although there is increasing evidence of negative feedback loops of Wnt/β-catenin signaling^4,5^, how Wnt/β-catenin signaling is amplified and maintained by positive feedback regulation in colon cancer cells remains unclear. It is interesting to note that MACC1 expression is positively correlated with β-catenin expression in CRC tissues and that MACC1 overexpression in colon cancer cells promotes migration, invasion, and tumor formation through stimulating β-catenin and its target gene expression^23^. These observations, together with our findings that MACC1 is a direct transcriptional target of β-catenin, suggest a positive feedback circuit in which β-catenin promotes the expression of MACC1, which then potentiates Wnt/β-catenin signaling in colon cancer cells.

As a transcriptional activator, MACC1 directly regulates the transcription of numerous genes involved in EMT, metastasis, cancer stemness, and drug resistance, including MET, SPON2, and MDR1/ABCB1^15–18^. Loss of DBC1 results in deregulation of MACC1 target gene expression, and DBC1 is required for cell proliferation, EMT properties, CSC-like traits, and drug resistance potential of colon cancer cells. These results indicate the importance of DBC1 for the expression of MACC1 and its target genes, which promote colon cancer progression and stimulate the expansion of CSC-like cells and might also suggest a functional interaction between MACC1 and DBC1. It would be of interest to explore the possibility that DBC1 can function as a coactivator for MACC1-mediated transactivation.

Our recent study identified DBC1 as a key regulator of β-catenin-PROX1 transcriptional axis and showed a positive correlation between DBC1 expression and poor outcome in advanced CRC^9^. Here we provide new insights into the role of DBC1 in colon cancer progression. Our findings imply that, in addition to stimulating Wnt/β-catenin-mediated MACC1 expression, DBC1 may also contribute to a more aggressive phenotype of colon cancer by promoting MACC1-mediated transcription in colon cancer cells. Thus, DBC1 is not only a pivotal regulator of CRC progression through acting as a tumor-promoting coactivator in multiple transcriptional pathways activated by Wnt/β-catenin signaling, but also a prognostic and therapeutic target for CRC.

## Materials and methods

### Quantitative real-time reverse transcription-PCR and ChIP-qPCR

Total RNA was extracted from cultured cells using Trizol (Invitrogen) or from formalin-fixed, paraffin-embedded xenograft tumor sections using RNeasy FFPE Kits (Qiagen), and qRT-PCR was performed with Brilliant II SYBR Green QRT–PCR Master Mix 1-Step (Agilent). ChIP and ChIP-qPCR experiments were performed as previously described^8–10,41^. The primers used in qRT-PCR and ChIP-qPCR are listed in Supplementary Information.

### DNA affinity precipitation assays

DAPA was performed as described previously^10,42^. Briefly, the MACC1 enhancer region was amplified from the MACC1.enh-TA-LUC with biotinylated PCR primers and incubated with SW480 nuclear extracts or 293T cell extracts transfected with LEF1, β-catenin, and DBC1 expression vectors. Probe-bound proteins were collected with streptavidin agarose beads (ThermoFisher Scientific) and analyzed by immunoblot.

### 3C

The 3C assays were carried out as previously described^9,10,42^. Briefly, cross-linked chromatin from SW480 or HT-29 cells was digested with BsrGI and ligated with T4 DNA ligase under diluted conditions. After reversing cross-links, the purified 3C DNA was analyzed by PCR. 3C and input PCR primers are listed in Supplementary Information.

### Colonosphere formation and extreme LDA

A total of 1 × 10^2^ cells/well of HT-29 or 2 × 10^2^ cells/well of SW480 were seeded in ultra-low attachment 96 well plate (Costar). Colonospheres were cultured in serum-free sphere media (DMEM/F12 supplemented with 20 ng/ml hEGF, 10 ng/ml bFGF, 1x N-2, and 1x B-27) for 6–10 days, and the number of colonospheres with a diameter of >40 μm was counted. To induce differentiation, an aliquot of spheroid HT-29 cells was trypsinized and cultured in DMEM with 10% fetal bovine serum. For oxaliplatin sensitivity assay, FLAG or FLAG-DBC1-expressing colonospheres were cultured in the presence of 10 µM oxaliplatin (Sigma-Aldrich). For extreme LDA, HT-29 or SW480 cells were plated in ultra-low attachment 96 well plates in sphere media at a density that ranged from 80 to 10 cells/well (HT-29) or ranged from 400 to 50 cells/well (SW480) (six replicates/cell concentration). After 6–10 days sphere culture, the number of wells containing colonospheres was counted, and the frequency of sphere-forming cells was determined using the Extreme Limiting Dilution Analysis software (http://www.bioinf.wehi.edu.au/software/elda/)^43^.

### Immunofluorescence staining of colonospheres

HT-29 cells infected with lentivirus expressing FLAG or FLAG-DBC1 were placed on cover slips coated with poly-D-lysine, and immobilized colonospheres were fixed with 4% paraformaldehyde and permeabilized with methanol. After incubation with blocking buffer (10% normal goat serum, 100 mM glycine, and 0.1% Triton X-100 in PBS), colonospheres were incubated with anti-MACC1 (1:200), washed, and subsequently incubated with Alexa Fluor 488-labeled second antibodies (ThermoFisher Scientific). Nuclei were stained with ProLong Gold Antifade Mountant with DAPI (ThermoFisher Scientific). Confocal fluorescent images were obtained using a Zeiss LSM 700 laser scanning confocal microscope (Carl Zeiss).

### Bioinformatics and statistical analysis

The ChIP-seq datasets of TCF4/TCF7L2 (wgEncodeEH002022), H3K4Me1 (wgEncodeEH002874), and H3K27Ac (wgEncodeEH002873) were obtained from the ENCODE data repository site (http://genome.ucsc.edu/ENCODE/). The ChIP-seq peaks were visualized with the UCSC genome browser and the Integrative Genome Viewer (IGV, http://www.broadinstitute.org/software/igv/). R2 Genomics Analysis and Visualization platform (http://r2.amc.nl) was used to investigate the correlation between DBC1 and MACC1 mRNA expression in three colon cancer microarray datasets (GSE75315, GSE21510, and e-metab-990). SurvExpress (http://bioinformatica.mty.itesm.mx/SurvExpress) was used to determine prognostic values of DBC1 and MACC1 mRNA expression and provide their risk assessment in colon cancer. Kaplan-Meier survival analyses were performed with publicly available colon cancer datasets (GSE28722 and GSE24551). Statistical significance (*P* < 0.05) for qRT-PCR, ChIP-qPCR, FAIRE, sphere formation, cell proliferation, migration, and invasion experiments was assessed by Student’s two-tailed *t*-test.

## Electronic supplementary material

Supplementary Information
