# Supplementary material for: DBC1 regulates Wnt/β-catenin-mediated expression of MACC1, a key regulator of cancer progression, in colon cancer
Source: Cell Death Dis. 2018 Aug 6;9(8):831. doi: 10.1038/s41419-018-0899-9 (PMC6079074; doi:10.1038/s41419-018-0899-9)
Supplement: Supplementary file 1 — Supplementary Information [file 41419_2018_899_MOESM1_ESM.pdf]

Supplementary Information

Supplementary Materials and Methods

Cell culture and transfection

HT-29, SW480, 293T, and L-Wnt-3a cells were maintained in DMEM with 10% fetal bovine serum. SW480 DBC1 knockout (KO) cells were described previously[1]. All cell lines were obtained from the American Type Culture Collection (ATCC), authenticated by short tandem repeat profiling, and routinely tested for mycoplasma-negative. For transient transfection and reporter gene assays, cells were transfected with expression plasmids using Lipofectamine 3000 reagent (ThermoFisher Scientific) and treated with 20 mM LiCl, Wnt3a-CM, and 50 μM iCRT14 (Santa Cruz Biotechnology). Wnt3a-CM was prepared by collecting the culture supernatant of L-Wnt-3a cells stably expressing Wnt3a according to the ATCC instructions.

Plasmids, RNA interference, and antibodies

The following previously described plasmids were used: pSG5.HA-β-catenin, pSG5.HA-LEF1, pSG5.HA-DBC1, pHR.CMV.puro.Sin8-shNS, pLKO.1-shDBC1#3, pLKO.1-shDBC1#8, pLKO.1-shβ-catenin#3, pHR.CMV.puro.Sin8-shβ-catenin (shβ-catenin#6), and pHR.CMV.FLAG.IRES-Hygro[1-5]. Lentiviral vector expressing shβ-catenin#5 (pLKO.1-shβ-catenin#5) was purchased from Sigma-Aldrich (TRCN0000003843). The MACC1.enh-TA-LUC reporter was constructed by cloning the MACC1 enhancer sequence (nucleotides +18,794 to +19,185 relative to transcription start site) into pTA-LUC (Clontech). MACC1 cDNA, PCR-amplified from SW620 cells, was cloned into pSG5.HA. DBC1 cDNA was cloned into the lentiviral vector pHR.CMV.FLAG-IRES-Hygro. Depletion of DBC1 or β-catenin using shRNAs or siRNAs was performed as described previously[1,4,5]. Non-specific (NS) siRNA and siRNAs for DBC1 (siDBC1#1 and siDBC1#2) were described previously[4]. The MACC1 shRNA expression lentiviral vector (pLKO.1-shMACC1) was constructed by inserting the following annealed oligonucleotides into pLKO.1 (Sigma-Aldrich): shMACC1, 5'-CCG G<u>GA TTG GAC TTG TAC ACT GC</u>T TCA AGA GA<u>G CAG TGT ACA AGT CCA ATC</u> TTT TTG-3' (sense) and 5'-AAT TCA AAA A<u>GA TTG GAC TTG TAC ACT GC</u>T CTC TTG AA<u>G CAG TGT ACA AGT CCA ATC</u>-3' (antisense). Lentiviruses expressing shRNAs or overexpressing DBC1 were produced as described previously[1,2].

Primary antibodies used in this study are as follows: anti-MACC1 HPA020103 (HPA); anti-β-catenin H-102 and anti-LEF1 H-70 (Santa Cruz Biotechnology); anti-TCF4 05-511 (Millipore); anti-DBC1 434A (Bethyl Laboratories) and GTX49199 (Genetex); anti-acetyl-histone H3 antibody 06-599 (Millipore); anti-GAPDH LF-PA0018 (AbFrontier); anti-HA 3F10 (Roche).

Gene set enrichment analysis (GSEA)

GSEA was performed as described previously using GSEA program (http://www.broadinstitute.org/gsea/) with MACC1 target gene sets (top 100 differentially expressed genes between control and MACC1-overexpressing SW480 cells) and DBC1 target gene sets (1433 differentially expressed genes between control and DBC1 KO SW480 cells)[1,6].

FAIRE

FAIRE assays were carried out as previously described[7,8], and FAIRE-enriched chromatin was analyzed by qPCR with ChIP PCR primers for the MACC1 enhancer.

Cell proliferation, migration, and invasion assays

Cell proliferation, migration and invasion assays were carried out as previously described[1].

Immunohistochemistry

Immunohistochemical staining of DBC1 were performed as described previously[1]. SW480 xenograft tumors were obtained from our previous in vivo study[1]. Formalin-fixed paraffin-embedded xenograft tumor sections were deparaffinized, rehydrated, and subjected to antigen retrieval. The tumor sections were incubated with anti-DBC1 antibody, washed, and then incubated with a horseradish peroxidase-labelled secondary anti-rabbit antibody (EnVision Detection System, Dako). Digital images of tissue sections were captured using the Aperio ScanScope XT slide scanner (Aperio Technologies).

Primer sets used in this study

qRT-PCR was performed using following forward (F) and reverse (R) primers (5' to 3'): MACC1, TCT GTA TGA ACT TAT TGT GGC TC (F) and CAT AGG CAG GTT TCC ACA TC (R); DBC1, AAG GTG CAA ACG CTC TCC AAC CAG (F) and GGA TGT TTG GAA

GAG ACT CAG AG (R); β-catenin, ACA AGA AAC GGC TTT CAG TTG AGC (F) and TCC TGG CCA TAT CCA CCA GAG T (R); β-actin, CCA CAC TGT GCC CAT CTA CG (F) and AGG ATC TTC ATG AGG TAG TCA GTC AG (R); GAPDH, TCT GGT AAA GTG GAT ATT GTT GCC (F) and GAA GAT GGT GAT GGG ATT TCC (R); MET, CAT GCC GAC AAG TGC AGT A (F) and TCT TGC CAT CAT TGT CCA AC (R); SPON2, TGG TCT CGT TTG TGG TGC (F) and GGA GGA CGT TAT CTC GGT CA (R); ABCB1, GGG AGC TTA ACA CCC GAC TTA (F) and GCC AAA ATC ACA AGG GTT AGC TT (R); GPX2, GAC TTC ACC CAG CTC AAC GAG (F) and GAT GCT CGT TCT GCC CAT TCA (R); ANK3, GGA GCA TCA GTT TGA CAG CA (F) and TTC CAC CTT CAG GAC CAA TC (R); CCND2, GAG AAG CTG TCT CTG ATC CGC A (F) and CTT CCA GTT GCG ATC ATC GAC G (R); TMPRSS2, CCT GCA GGG ACA TGG GCT ATA (F) and CCG GCA CTT GTG TTC AGT TTC (R).

ChIP-qPCR was performed using following primers (5' to 3') (nucleotides relative to transcription start site): MACC1 Enhancer (Intron 1 WREs, +18,822~+19,056), GAA TAA TCA AAG GGT GGA GCC TGG (F) and GGC ATC AGA TCC ACA GAC AGC ATG (R); MACC1 intron 2 (a negative control region, +48,211~+48,371), GGC AAC AGT GGG GAT TAG GTA (F) and TGT GCC ATG ATA GGG ACT GCA (R).

3C PCR was performed using following primers (5' to 3') (nucleotide position relative to transcription start site): MACC1 3C P+ (+808), CCT GAG GTA TCT GGA GTA GTC AAA CT; MACC1 3C E- (+17,457), ACC AAG TTT TAA GGC TCA AAT GAA or (+17,430) GCC AAA TCC TAT ACA ATT ATT AAC AAG ATA; Input C+ (+18,334), CAT TCT CCT TAC ATA CTG GCA GCT G; Input C- (+18,631), CCA TGC ACC ACA AGG AGA CT.

The primers used for cloning of the MACC1 enhancer (Intron 1 WREs, nucleotides +18,794 to +19,185 relative to transcription start site): AAT ACG CGT CAG ACC AGG AGC TTA TCA (F) and TGG CTC GAG AAG CAA ACC ATT ACC (R) (MluI and XhoI sites for cloning are underlined).

The primers used to amplify probes for DAPA (MACC1 Intron 1 WREs, nucleotides +18,794 to +19,185 relative to transcription start site): 5'-Biotin-CAG ACC AGG AGC TTA TCA AGA (F) and TGG CTC GAG AAG CAA ACC ATT ACC (R)

Supplementary References

1 Yu, E. J. *et al.* Positive regulation of beta-catenin-PROX1 signaling axis by DBC1 in

colon cancer progression. *Oncogene* **35**, 3410-3418 (2016).
2 Kim, J. H. *et al.* CCAR1, a key regulator of mediator complex recruitment to nuclear receptor transcription complexes. *Mol Cell* **31**, 510-519 (2008).
3 Kim, H. J., Kim, S. H., Yu, E. J., Seo, W. Y. & Kim, J. H. A positive role of DBC1 in PEA3-mediated progression of estrogen receptor-negative breast cancer. *Oncogene* **34**, 4500-4508 (2015).
4 Yu, E. J. *et al.* Reciprocal roles of DBC1 and SIRT1 in regulating estrogen receptor α activity and co-activator synergy. *Nucleic Acids Res* **39**, 6932-6943 (2011).
5 Ou, C. Y. *et al.* A coactivator role of CARM1 in the dysregulation of beta-catenin activity in colorectal cancer cell growth and gene expression. *Mol Cancer Res* **9**, 660-670 (2011).
6 Schmid, F. *et al.* SPON2, a newly identified target gene of MACC1, drives colorectal cancer metastasis in mice and is prognostic for colorectal cancer patient survival. *Oncogene* **35**, 5942-5952 (2016).
7 Moon, S. J. *et al.* DBC1 promotes castration-resistant prostate cancer by positively regulating DNA binding and stability of AR-V7. *Oncogene* **37**, 1326-1339 (2018).
8 Seo, W. Y. *et al.* CCAR1 promotes chromatin loading of androgen receptor (AR) transcription complex by stabilizing the association between AR and GATA2. *Nucleic Acids Res* **41**, 8526-8536 (2013).

Supplementary Figures

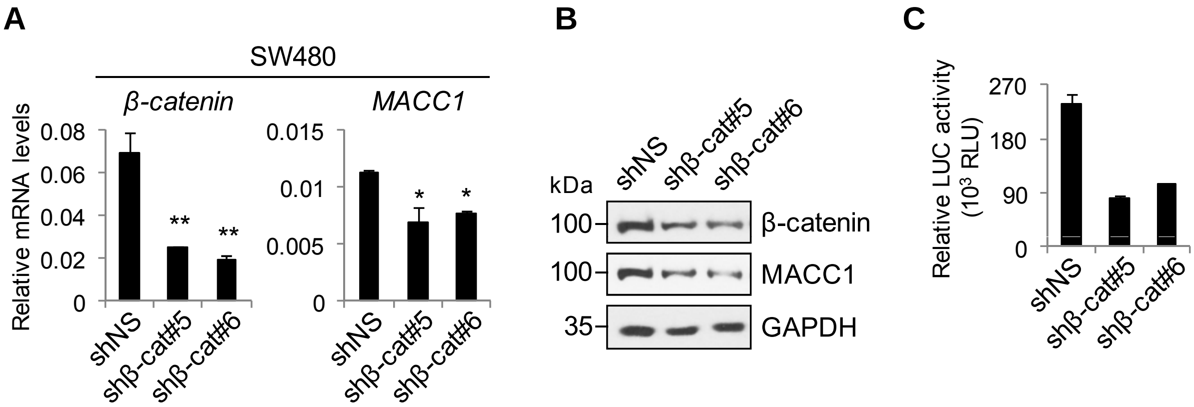

Supplementary Figure S1. β-catenin is required for MACC1 expression. To rule out the possible off-target effects, we used two additional shRNAs targeting different regions of β-catenin mRNA. SW480 cells were infected with lentiviruses expressing shNS, shβ-catenin#5, or shβ-catenin#6. The mRNA and protein levels of β-catenin and MACC1 were examined by qRT–PCR (A) and immunoblot (B). Data are means±s.d. (n=3). *P<0.05 and **P<0.01. (C) MACC1.enh-TA-LUC reporter activity in SW480 cells infected with lentiviruses expressing shNS, shβ-catenin#5, or shβ-catenin#6. Data are means±s.d. (n=3).

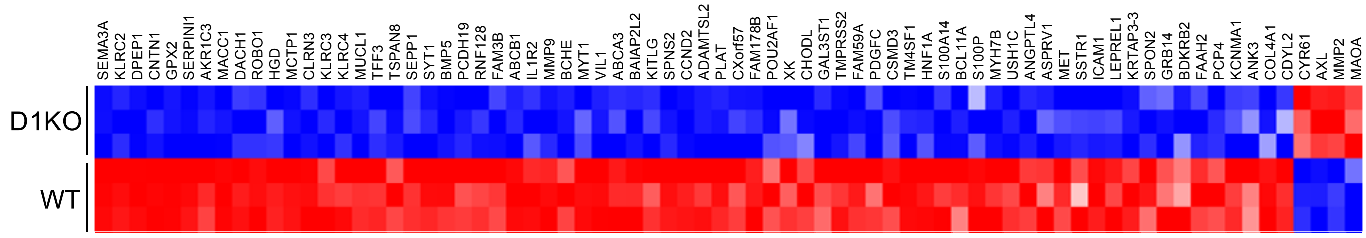

Supplementary Figure S2. Heat map of DBC1-dependent MACC1 target genes.

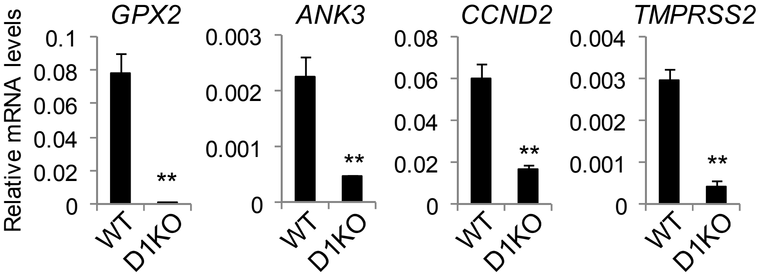

Supplementary Figure S3. Validation of DBC1-regulated MACC1 target genes. Total RNAs from SW480 WT and DBC1 KO cells were examined by qRT–PCR analysis with primers specific for the indicated mRNAs. Data are means±s.d. (n=3). **P<0.01.

+808 CCTGAGGTATCTGGAGTAGTCAAACTCACATAAATGATACTTAGAATGGTAGTTGCCAGGGGCTTG
GGGGAAGGAGAAATGAGAGTTGTTTAATGGATATAGAGTTTCAGTTTACAAGATGAAAACACTTGG
AGAC<u>TGTACA</u>CACACACACACACACACATAATGGTATAGCCACTTGTTTATCTTTATTCTCTATCT
TGTTAATAATTGTATAGGATTTGGCTAATTCATTTGAGCCTTAAAACTTGGT +17457

Supplementary Figure S4. Sequence of the 250 bp 3C product. The ligated BsrGI site is indicated by underline.

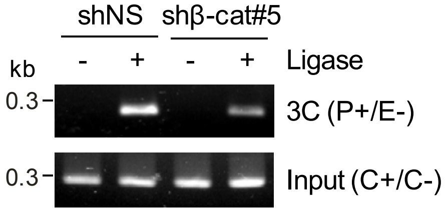

Supplementary Figure S5. 3C experiments. Cross-linked chromatins from SW480 cells expressing shNS or shβ-catenin#5 were digested with BsrGI, diluted, and ligated. After reverse-crosslinking, equal amounts of 3C DNA were amplified by PCR using primer pairs P+ and E- (3C) or C+ and C- (Input).

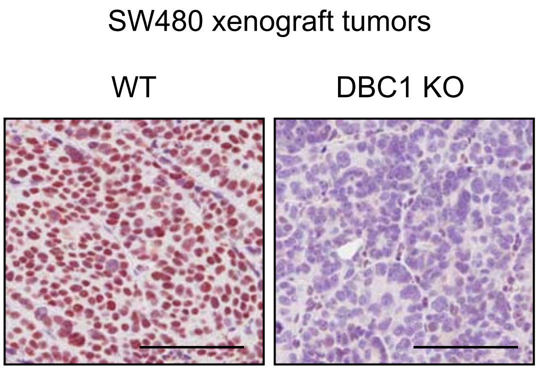

Supplementary Figure S6. Representative images showing levels of DBC1 immunostaining in SW480 xenograft tumors. Scale bar: 100 µm.

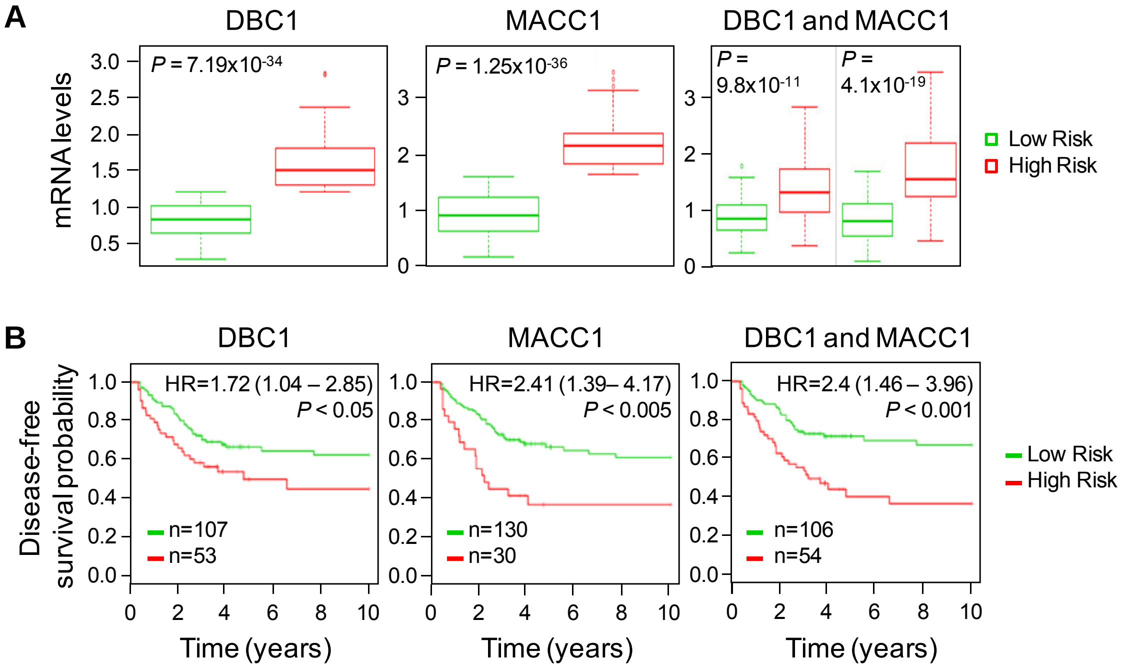

Supplementary Figure S7. DBC1 and MACC1 correlate with poor prognosis of colon cancer patients. Box-plots and Kaplan-Meier survival curves were generated with SurvExpress biomarker validation tool using a colon cancer dataset (GSE24551). Expression levels of DBC1, MACC1, and both gene stratified by risk groups (A) and Kaplan-Meier survival curves of Cox analysis for the colon cancer dataset stratified by maximized DBC1, MACC1, and both gene expression risk groups (B).
